# Parochial Beds

**Published:** 1924-10

**Authors:** 


### PAROCHIAL BEDS.

The difficulty of organising hospital funds in large towns is proverbial. In villages and small towns the individual can easily be interested, but a very large organisation would be required for this in great towns, and even then many householders would be overlooked. In the great appeal that has been made at Leicester, Mr. Harry Johnson, the house governor of the Royal Infirmary, discovered that while the county raised the sum for which it was asked, the city subscribed nearly one-third less than was hoped. One of the methods adopted in the county was for different parishes to endow beds. Mr. Johnson has reminded the city that its churches raised last Hospital Sunday enough to maintain two wards, with twelve beds apiece. But this fund is not supported by all the churches. It has therefore been suggested that the city parishes should adopt the county plan, and either endow or maintain a bed or beds according to their capacities. The idea is worth considering for it would require more than a Sunday collection to do this, and consequently interest would be maintained for a definite object. Any plan which promises some solution of the problem of hospital maintenance in cities is worth trying, and we hope this one will be given a fair trial.

